# Ultrahigh-density spin-polarized hydrogen isotopes from the photodissociation of hydrogen halides: new applications for laser-ion acceleration, magnetometry, and polarized nuclear fusion

**DOI:** 10.1038/s41377-021-00476-y

**Published:** 2021-02-12

**Authors:** Alexandros K. Spiliotis, Michalis Xygkis, Michail E. Koutrakis, Konstantinos Tazes, Gregoris K. Boulogiannis, Chrysovalantis S. Kannis, Georgios E. Katsoprinakis, Dimitrios Sofikitis, T. Peter Rakitzis

**Affiliations:** 1 Foundation for Research and Technology Hellas, Institute of Electronic Structure and Laser, N. Plastira 100, Heraklion, Crete GR-71110 Greece; 2 University of Crete, Department of Physics, Herakleio, Greece; 3 Department of Physics, Atomic and Molecular Physics Laboratory, University of Ioannina, University Campus, Ioannina, GR-45110 Greece

**Keywords:** Plasma-based accelerators, Laser-produced plasmas, Imaging and sensing

## Abstract

Recently, our group produced spin-polarized hydrogen (SPH) atoms at densities of at least 10^19^ cm^−3^ from the photodissociation of hydrogen halide molecules with circularly polarized UV light and measured them via magnetization-quantum beats with a pickup coil. These densities are approximately 7 orders of magnitude higher than those produced using conventional methods, opening up new fields of application, such as ultrafast magnetometry, the production of polarized MeV and GeV particle beams, such as electron beams with intensities approximately 10^4^ higher than current sources, and the study of polarized nuclear fusion, for which the reaction cross sections of D–T and D–^3^He reactions are expected to increase by 50% for fully polarized nuclear spins. We review the production, detection, depolarization mechanisms, and potential applications of high-density SPH.

## Spin-polarized hydrogen: production by photodissociation

A laser-based scheme to produce spin-polarized hydrogen (SPH) at much higher densities than conventional methods could create new opportunities in physics. In a review of the topic, Alexandros Spiliotis and coworkers from the University of Create and FORTH Institute of Electronic Structure and Laser in Greece describe how the use of circularly polarized, pulsed ultraviolet laser light can produce SPH with a density of 10^19^ cm^-3^ by photodissociation of hydrogen halide molecules. This high-density production approach brings many benefits including the prospect of ultrafast magnetometry and the generation of high energy beams of spin-polarized electrons, protons or deuterons by laser-plasma acceleration. Furthermore, spin-polarized nuclear fusion is expected to be more efficient than conventional fusion.

## Introduction

The production and manipulation of spin-polarized electrons and nuclei are fundamental to many fields of physics, including nuclear magnetic resonance (NMR), electron-spin resonance (ESR), magnetometry, spintronics, Bose–Einstein condensation (BEC), and in studying scattering and reactions in atomic, molecular, optical, particle, and nuclear physics^1–7^. However, the equilibrium polarization of a sample, for all but cryogenic temperatures, is very small (typically less than 10^−5^, even for large magnetic fields of approximately ∼1 T). Therefore, the key to producing large polarizations in environments with rapid depolarization mechanisms is to polarize rapidly in situ (if possible) or to polarize in conditions where depolarization is small, then to quickly introduce the polarized matter into the desired environment where the depolarization rates are high and to perform the desired experiments rapidly, before significant depolarization occurs. One example of this is the introduction of spin-polarized ^129^Xe into the human body to enhance magnetic resonance imaging (MRI) signals for diagnostic purposes^8^. Thus, the time for the production of spin polarization, particularly in situ, is of paramount importance for applications of polarized matter.

Recently, our group produced spin-polarized hydrogen (SPH) and deuterium (SPD) atoms at densities of at least 10^19^ cm^−3^ and on the 100 ps timescale from the photodissociation of hydrogen halides^9^. These densities and timescales are at least 7 orders of magnitude higher and faster, respectively, compared to conventional production methods of SPH. These new regimes of SPH density and near-instantaneous in situ polarization production open up a range of new applications of SPH. The aim of this review is to describe the production, detection, and depolarization of SPH in this ultrahigh-density regime and to describe the new applications that are now possible. Specifically, in section II, we provide the details of the photodissociation of hydrogen halides and explain why the SPH density can surpass conventional SPH production techniques. In section III, we describe the detection of SPH via the measurement of magnetization-quantum beats with a pickup coil. In section IV, we describe the depolarization mechanisms that limit the SPH lifetime, both for low-density SPH and for high-density SPH. Finally, the last three sections deal with new applications: section V describes fast magnetometry, with ns time resolution; section VI describes the production of spin-polarized electron and proton beams from laser-plasma acceleration, with the potential to surpass the flux of current sources by 4 orders of magnitude^10^; and section VII describes proposals for testing polarized fusion, for the D–T and D–^3^He reactions, where it is expected that polarizing the nuclear spins will increase the fusion cross section by 50%; until now, such tests have not been performed in a plasma due to the lack of sources that can produce SPD at sufficiently high density.

## SPH production

The conventional methods for SPH production are the Stern–Gerlach spin-separation technique (or atomic beam source (ABS)) and spin-exchange optical pumping. We first describe these methods in some detail.

### Atomic beam source

An ABS produces an atomic beam of unpolarized hydrogen atoms, which then passes through an inhomogeneous magnetic field, which induces a deflection force on the spin-up electrons and an opposite force on the spin-down electrons. These forces allow the separation of the atoms with spin-up and spin-down electrons into separate beams. However, the time and distance needed for this beam separation are very long: the distance is on the order of 1 m for a few mm of beam separation, and the separation time is on the order of 1 ms. The density of the beam must be kept below a critical limit, above which velocity-changing collisions between atoms diffuse the atomic beam to the point where spin separation is no longer spatially resolved. The critical density limit is approximately 10^12^ cm^−3^, and a typical beam velocity of ∼2000 m/s and a beam cross-sectional area of ∼0.1 cm^2^ yield an SPH production rate of nearly 10^17^ s^−1^ ^11^. We note that these densities (less than 10^−7^ bar) and production rates (less than 1 μmol s^−1^) are microscopic and limit the range of application significantly.

### Spin-exchange optical pumping (SEOP)

Optical pumping is a method for transferring polarization from photons to atoms for atoms with strong transitions that can be pumped with powerful lasers. The ground-state H atom’s first transition is at 121.6 nm in vacuum UV light, which is not convenient for optical pumping (strong lasers do not exist there, among many other problems). The alkali atoms have strong transitions in the near IR and can be excited with circularly polarized σ^+^ radiation, which increases the angular momentum projection of the excited-state atom by one unit of *ℏ*. The atom then fluoresces, and some fraction of this polarization is retained, on average, in the ground state. This polarization cycle (optical excitation and fluorescence) needs to be repeated many times (approximately 10) until the atomic polarization (nuclear and electronic spin) approaches 100%, and when performed with cw lasers, the polarization of the alkali atoms can be maintained continuously at a density of approximately 10^13^ cm^−3^. While these high-density polarized alkali atoms do not have any direct application (beyond fundamental research), they can be used as a polarization source to transfer polarization to other atoms through collisions^12,13^, termed SEOP. SEOP has been used to produce SPH at production rates of approximately 10^17^ s^−1^, similar to ABS. The main application of SEOP is the production of spin-polarized noble gases with nuclear spins (mainly ^3^He and ^129^Xe) in macroscopic quantities (∼10 L bar/h), which are used for MRI signal enhancement. The success with ^3^He and ^129^Xe is mainly due to the very slow depolarization rates of these noble gases, as the nuclei are protected from depolarization due to the closed-shell electronic structure. However, similar to ABS, the polarization time for SEOP is very slow and cannot polarize open-shell atoms at high densities.

### SPH from photodissociation

The production of spin-polarized atoms from diatomic molecule photodissociation has significant similarities to both ABS and SEOP but also important differences. We first provide details on the photodissociation mechanism before drawing these comparisons.

Spin-polarized atoms can be produced from the photodissociation of diatomic molecules with circularly polarized light^14–16^ because the electronic angular momentum projection of a particular molecular state adiabatically correlates to particular m states of the separated atoms^17,18^. In particular, the total electronic angular momentum projection quantum number Ω is equal to the sum of the m states of the separated atoms m_A_ and m_*B*_:

1 $${\Omega} = m_A + m_B$$

**Fig. 1:**
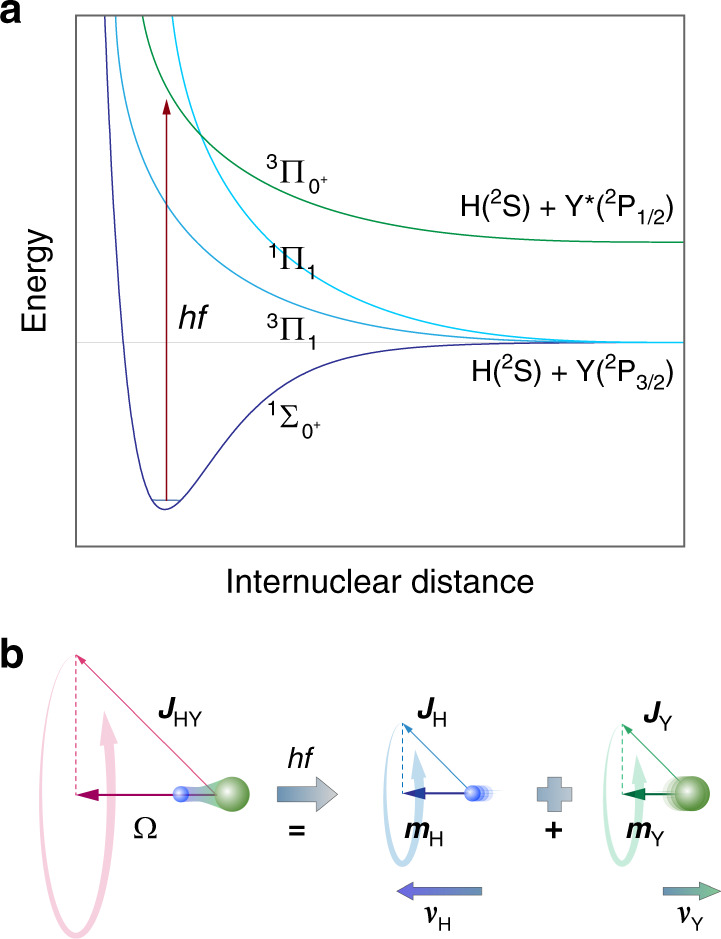
Correlation of molecular electronic states to atomic m states. **a** The potential energy curves that participate in the photodissociation HY. **b** The total electronic angular momentum projection (Ω) of HY is conserved during the dissociation and is equal to the sum of the product m states: Ω = m_*A*_ + m_*B*_

Hydrogen halides, HY, have been shown to be the most effective molecules for the production of SPH^19–29^, as they are the only diatomic molecules that contain H atoms except for H_2_ (which photodissociates below 100 nm, where high photon fluxes cannot be produced). The potential energy curves of the three lowest electronic states of HY that play a role in the photodissociation and photofragment polarization are shown in Fig. 1, *A* ^1^Π_1_, *a* ^3^Π_1_, *a* ^3^Π_0+_, as well as the ground state X ^1^Σ_0+_ ^30–33^. These three states correlate adiabatically to the atomic m states as

2 $$\begin{array}{l}{\mathrm{HY}}(A^1\,\prod _1;\,{\Omega} = \pm 1) \to {\mathrm{H}}({\mathrm{m}}_{\mathrm{H}} = \mp 1/2)\\ + {\mathrm{Y}}({\mathrm{m}}_{\mathrm{Y}} = \pm 3/2)\end{array}$$

3 $$\begin{array}{l}{\mathrm{HY}}(a^3\,\prod _1;\,{\Omega} = \pm 1) \to {\mathrm{H}}({\mathrm{m}}_{\mathrm{H}} = \pm 1/2)\\ + {\mathrm{Y}}({\mathrm{m}}_{\mathrm{Y}} = \pm 1/2)\end{array}$$

4 $$\begin{array}{l}{\mathrm{HY}}(a^{3}\, \prod _{0 +};\,{\Omega} = {0})\, {\to}\, {\mathrm{H}}({\mathrm{m}}_{\mathrm{H}} = {\pm} 1/2)\\ + {Y}^{\ast} ({\mathrm{m}}_{{\mathrm{Y}}^{\ast}} = {\mp} 1/2)\end{array}$$

where Y refers to ground-state Y(^2^P_3/2_) atoms and Y* refers to spin–orbit-excited Y(^2^P_1/2_) atoms.

For maximum polarization and for ease of understanding, the molecular bonds can be aligned along the polarization direction of the photodissociation laser using the method of strong-field laser alignment. For such bond-aligned molecules and for a circularly polarized σ^+^ photodissociation laser, the allowed transitions are from the ground state Ω = 0 to the Ω = + 1 *A* ^1^Π_1_ and *a* ^3^Π_1_ states, as well as to the Ω = 0 *a* ^3^Π_0+_ state. We observe, from Eqs. (2–4), that, for adiabatic dissociation, the *A* ^1^Π_1_ state yields H atoms with electrons that are spin down, the *a* ^3^Π_1_ state yields H atoms with electrons that are spin up, whereas the *a* ^3^Π_0+_ state yields H atoms with electrons that are unpolarized. Therefore, one can produce H atoms with 100% spin-polarized electrons, from excitation to the *A* ^1^Π_1_ *or a* ^3^Π_1_ states, followed by adiabatic dissociation (but not from exciting a superposition of the *A* ^1^Π_1_ and *a* ^3^Π_1_ states). Excitation of the ^3^Π_0+_ state (which yields unpolarized H atoms) is avoided if the molecular bonds are aligned parallel to the propagation direction of the circularly polarized photodissociation laser.

For all hydrogen halide (HY) molecules, it is possible to excite, almost exclusively, the *A* ^1^Π_1_ state, or the *a* ^3^Π_1_ state, with a large absorption cross section in the UV. For example, HI can be photodissociated almost exclusively through the *A* ^1^Π_1_ state at 213 nm and through the *a* ^3^Π_1_ state at 266 nm. Therefore, conditions exist where the excitation step is fully compatible with the production (near) of 100% spin-polarized H atoms.

Nonadiabatic transitions during dissociation can also occur, which is an additional potential complication. These nonadiabatic transitions transfer the population from one state to another at long interatomic distances, and they involve spin flips in both atomic photofragments while maintaining the condition of Eq. (1). For example, after exclusive excitation to the *A* ^1^Π_1_ state (which adiabatically yields SPH with spin-down electrons), nonadiabatic transfer to the *a* ^3^Π_1_ state yields SPH with *spin-up* electrons. Clearly, a large mixture of adiabatic and nonadiabatic dissociation strongly reduces the spin polarization, and either (near-)complete adiabatic or nonadiabatic dissociation is desirable to produce highly spin-polarized H atoms. It would be useful if theoretical calculations could accurately predict the contribution of nonadiabatic transitions; while successful in the case of HCl, the contributions of nonadiabatic transitions were not treated properly for DI^34^. Fortunately, it has been shown experimentally that highly spin-polarized H or D atoms (with polarizations above 70%) are produced from photodissociation at wavelengths where powerful UV lasers exist: from HCl and HBr at 193 nm, from HCl, HBr, and DI at 213 nm, and from DI at 266 nm.

### Comparison of the photodissociation method to an ABS and SEOP

The photodissociation method involves both optical pumping and polarization through strong gradients of fields, so it can be compared directly to an ABS and SEOP. Optical pumping is relatively inefficient with the use of photons, as approximately 10 are needed to polarize the pumped atom, and many more are needed to maintain the polarization for the duration of the experiment. In contrast, the photodissociation method is maximally efficient, as it can produce SPH with an electron polarization of 100% with only one photon. The ABS spin separates a beam by having large magnetic-field gradients over a few cm and then accumulates a beam separation over approximately ∼1 m in ∼1 ms. The photodissociation method separates the energetic paths of the m-states of the atoms from the gigantic electric fields between the two nuclei over a distance of less than 1 nm and in ∼100 fs. The fact that the polarization time is approximately 9 orders of magnitude faster than that of the ABS suggests that the photodissociation method should operate at pressures approximately 9 orders of magnitude higher or at densities up to approximately 10^21^ cm^−3^; the observation of SPD in excess of 10^19^ cm^−3^ ^9^ and recent measurements of SPH at HCl densities of at least 10^20^ cm^−3^ ^35^ support this prediction. We describe the method for detecting these high SPH densities in the following section and the observed depolarization mechanisms in section IV.

## SPH detection

### SPH optical detection

SPH from photodissociation was first detected optically with two different methods. Before polarization-sensitive detection schemes for SPH became available, the SPH was measured indirectly: upon prompt photodissociation of HCl with circularly polarized light at 193 nm, both H and Cl atom cofragments are polarized, and the degree of H polarization can be inferred from the Cl polarization using Eq. (1). The polarization of the Cl atoms was measured using (2 + 1) resonance-enhanced multiphoton ionization (REMPI), using two photons to excite the Cl atoms to an intermediate state and one photon for ionization. The angular distribution of the Cl ion signal was measured with slice imaging, and the dependence of the angular distribution of the ions on the polarization directions of the photolysis and probe lasers provided sufficient information for the determination of the Cl polarization. The H atoms were inferred to have a polarization of approximately 72%^19^. Shortly afterwards, one of the authors proposed various polarized fluorescence methods to detect SPH directly^20^. This was achieved on the 1s-2p fluorescence transition at 121.6 nm^23,24^ and on the 2p-3d transition (followed by the two-photon 1s-3d excitation) at 656 nm^25^. The polarization of the fluorescence experiments agrees with the indirect REMPI experiments and with calculations. However, the optical detection methods are limited to low pressures of approximately 0.01 mbar (as the excited state is depolarized by collisions) and to low SPH densities of approximately 10^12^ cm^−3^ (at which point the optical transitions become optically thick, and light does not reach the detector). Therefore, it was not possible to optically measure SPH at high density to determine the upper limits possible with the photodissociation method. Crude estimates of the SPH depolarization cross sections indicated that SPH with a density of at least 10^16^ cm^−3^ may have a lifetime of >10 ns^22^, but these could not be confirmed optically.

### SPH pickup-coil detection

Samples with large time-dependent magnetization can be detected with a pickup coil, such as in NMR. However, the principle of detecting spins with a pickup coil, which has been produced with a short-pulsed laser, was demonstrated recently by Milner et al.^36^ from the production of spin-polarized O_2_ molecules from the centrifuging of O_2_ at ∼1 bar pressure with a tailored fs pulse. This demonstration inspired the measurement of SPH with a pickup coil^9^.

**Fig. 2:**
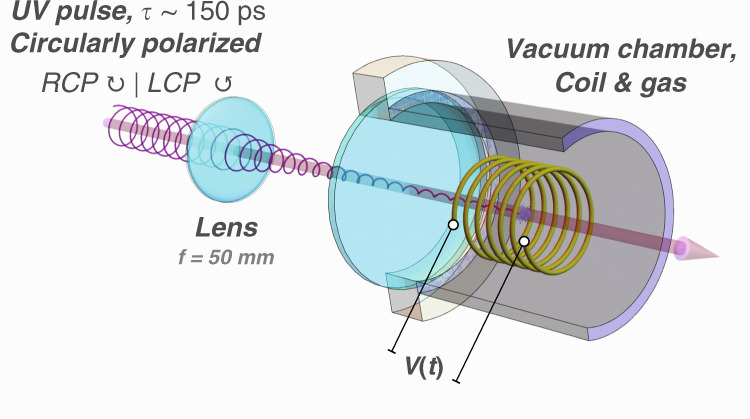
Experimental setup for the detection of SPH with a pickup coil. A circularly polarized, 150 ps UV pulse (at 213 or 266 nm) is focused into a vacuum chamber containing hydrogen halide gas at 0.1–5 bar, and passes through the pickup coil, and produces the signal *V*(*t*)

**Fig. 3:**
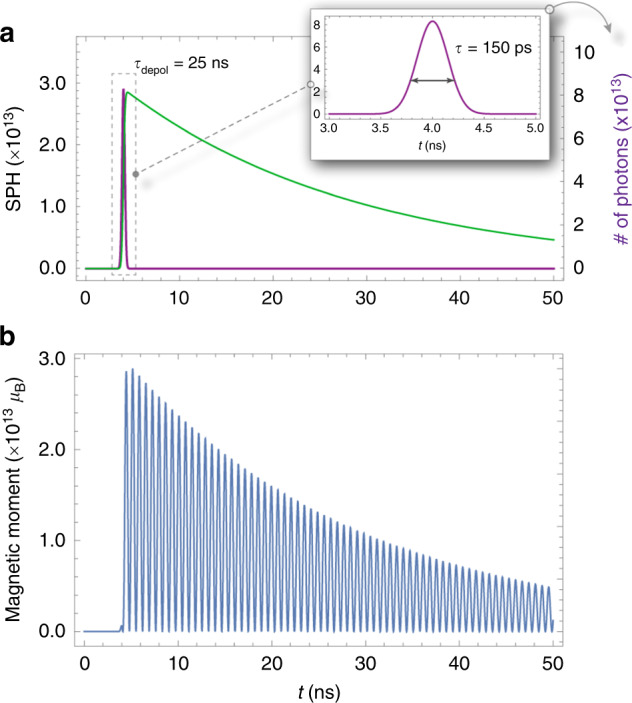
Time dependence of the electron-spin polarization. **a** The number of SPH (green curve—left axis) as a function of time after the arrival of the dissociation pulse (shown in the inset—representing the number of photons on the right axis). After the end of the pulse, the number of SPH are decaying exponentially due to depolarization mechanisms (here the depolarization time is 25ns). **b** The time-dependent electron magnetization, *M*(*t*), calculated using Eq. (5)

The experimental setup is shown in Fig. 2: a pickup coil with *N*∼4 turns, length *L*∼5 mm and diameter ∼2 mm is placed in a small vacuum chamber. A 150 ps, 213 nm or 266 nm, circularly polarized light pulse, with a pulse energy of 1–10 mJ, passes through the coil and photodissociates hydrogen halide gas at typical pressures of approximately 0.1–5 bar. For near optically-thick samples, up to approximately 10% of the light pulse can be absorbed in the coil, which corresponds to approximately 10^13^–10^15^ photons, producing 10^13^–10^15^ SPH atoms. The electrons of the H atoms are initially predominantly spin up, whereas the protons are unpolarized. The electron polarization is transferred to the protons and back at the hyperfine-beating frequency so that the electron magnetization *M(t)* is given by

5 $$M(t) = M_n{\rm{e}}^{ - t/\tau }{\rm{cos}}^2\left( {\frac{{\omega t}}{2}} \right)$$

where τ is the polarization lifetime, ω = (*E*_1_-*E*_0_)/ℏ is the angular hyperfine frequency, *E*_F_ are the energies of the hyperfine states *F* = 0 and 1, $M_n = g_Sm_SnP\mu _B \approx nP\mu _B$ is the magnetic moment of *n* spin-polarized electrons with initial polarization *P* (where −1 ≤ *P* ≤ 1), $\mu _B$ is the Bohr magneton, $g_S\approx2$ is the electron g factor, and *m*_*s*_=1/2 is the electron-spin projection. The exact form of *M(t)* is given by convoluting Eq. (5) with the pulse width of the photodissociation laser, as shown in Fig. 3.

**Fig. 4:**
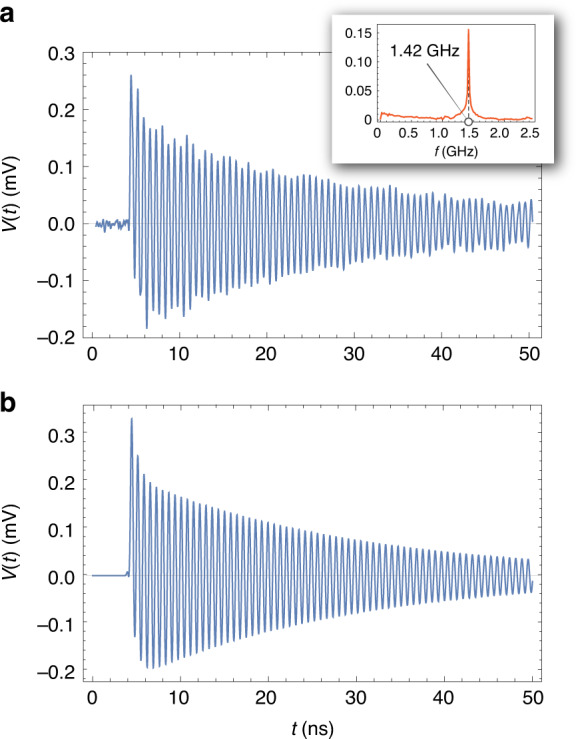
SPH magnetization-quantum-beat signals. **a** Experimental SPH magnetization-quantum-beat signal from the photodissociation of HBr at 75 mbar, with a lifetime τ = 25ns and beating frequency *f* = 1.42GHz, shown by the Fourier transform (inset), and **b** Theoretical trace calculated using Eq. (6) (see text for details)

The time-dependent magnetic flux created through the coil causes a time-dependent voltage in the coil *V(t)*. In simulating the detection apparatus, *V(t)* is treated as a time-dependent voltage source connected in series with a coil of inductance *L* (and with a negligible resistance) and a load resistor (the 50 Ω resistor of the 3 GHz oscilloscope (Rohde & Schwarz RTO2034) used in the experiment. The *V(t)* signal created in the coil, including that resulting from Lenz’s law, is the solution of the differential equation:

6 $$V(t) = \mu _0\frac{N}{l}\frac{{{\rm{d}}M(t)}}{{{\rm{d}}t}} - \frac{L}{R}\frac{{{\rm{d}}V(t)}}{{{\rm{d}}t}}$$

where *l* is the coil length and *N* is the number of coil turns. In Fig. 4a, an experimental magnetization-quantum beat signal is shown from SPH produced from the photodissociation of HBr at 75 mbar, with an exponential-decay lifetime τ_depol_ = 25 ns. The inset of Fig. 4a plots the Fourier transform of the experimental trace, depicting a strong peak at 1.42 GHz, the H atom hyperfine frequency. The theoretical trace, calculated by solving Eq. (6) and using values of τ_depol_ = 25 ns and *f* = 1.42 GHz, is shown in Fig. 4b.

Both signals resemble a damped oscillation; however, there are some more complicated features that are worth describing. First, both the theoretical and the experimental traces start with a rapid increase in *V(t)*, which relaxes soon (within two hyperfine periods). This rapid increase and its dynamics are related to the short laser pulse duration (150 ps) and are not related to the depolarization time τ at this pressure.

For negative *V(t)*, both the experimental and theoretical traces exhibit a smooth decrease before reaching the minimum value *V*_min_*(t)* ≈ −0.2 mV and subsequently follow the depolarization exponential decay. This slower decrease in the negative *V(t)* values is caused by the self-inductance of the detection coil. It is worth noting that by removing the second term in Eq. (3), this smooth decrease disappears from the theoretical trace, which becomes completely symmetric with respect to the x axis. We have seen, theoretically, that the effect of the detection-coil self-inductance on the signal gradually reduces for a lower frequency of the magnetization oscillation and becomes negligible below a few tens of MHz.

## SPH depolarization

We produce SPH from the photodissociation of HY at high pressure, typically between HY densities of 10^19^–10^20^ cm^−3^ (∼0.4–4 bar HY pressure). If the photodissociation laser is unfocused, then only approximately 0.1% of the HY is photodissociated in the laser beam, and SPH is produced at densities of ∼10^16^ cm^−3^, surrounded by nonphotodissociated HY at high density. If the photodissociation laser is focused, then essentially all the HY can be photodissociated near the laser focus, and the SPH density approaches the initial HY density of ∼10^19^–10^20^ cm^−3^. These two distinct density regimes, which we will call “low-density” and “high-density” SPH, show different depolarization mechanisms and will be discussed separately.

### Low-density SPH

**Fig. 5:**
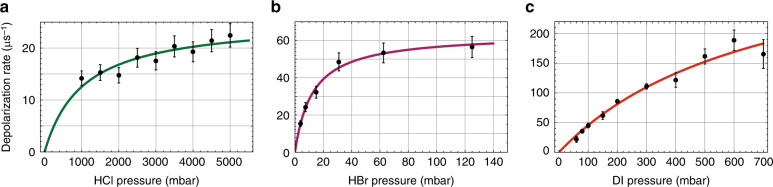
SPH depolarization rates. Measurements of the SPH depolarization rate as a function of hydrogen halide pressure^35^ for (**a**) HCl, (**b**) HBr, and (**c**) DI. The data are fit using Eq. (12)

We note that “low-density” SPH production, of order ∼10^16^ cm^−3^, is still approximately four orders of magnitude higher than that of conventional SPH production methods. The two possible depolarizers are Y atoms (at the same “low density” as SPH) and nonphotodissociated HY at “high density”, with bimolecular depolarization reactions:

7 $${\mathrm{H}}^ \uparrow + {\mathrm{Y}}\, {\mathop \rightarrow \limits^{k^Y}}\,{\mathrm{H}} + {\mathrm{Y}}$$

8 $${\mathrm{H}}^ \uparrow + {\mathrm{HY}}\,{\mathop \rightarrow \limits^{k^{{\mathrm{HY}}}}}\, {\mathrm{H}} + {\mathrm{HY}}$$

with rate constants *k*^Y^ and *k*^HY^, respectively. These depolarization reactions predict linear depolarization as a function of Y and HY density (Y density is proportional to HY density if the HY gas is not optically thick and limits the photodissociation light reaching the coil). However, the depolarization rates of SPH/SPD as a function of DI, HBr, and HCl pressure are not linear but show curved behaviour that asymptotes to a plateau, as shown in Fig. 5. The explanation for this behaviour has been the proposal of depolarization through an intermediate complex, HY-H^↑^, where the SPH polarization is transferred to the Y nucleus through the hyperfine interaction and is subsequently transferred to HY-H^↑^ rotation and depolarized by collisions. The reaction steps are given by the three reactions^35^:

9 $${\mathrm{H}}^ \uparrow + {\mathrm{HY}}\, \mathop { \rightleftharpoons }\limits_{{\mathrm{k}}_{{\mathrm{ - 1}}}}^{{\mathrm{k}}_1}\, {\mathrm{HY}} \cdots {\mathrm{H}}^ \uparrow$$

10 $${\mathrm{HY}} \cdots {\mathrm{H}}^ \uparrow \, {\mathop \rightarrow \limits^{k_d}} \, {\mathrm{HY}} \cdots {\mathrm{H}}$$

11 $${\mathrm{HY}} \cdots {\mathrm{H}}^ \uparrow + {\mathrm{HY}}\, {\mathop \rightarrow \limits^{k_2}} \, {\mathrm{H}}^ \uparrow + 2{\mathrm{HY}}$$

where Eq. (9) describes the complex formation and dissociation with rate constants *k*_1_ and *k*_−1_, respectively; Eq. (10) describes the nuclear hyperfine depolarization step, with rate constant *k*_d_, and Eq. (11) is the HY-H^↑^ dissociation from collisions with HY, with rate constant *k*_2_. Using the steady-state approximation and solving Eqs. (9–11) yields the SPH depolarization rate K=1/τ:

12 $$K = \frac{{k_1k_d\left[ {HY} \right]}}{{\left( {k_{ - 1} + k_d + k_2\left[ {HY} \right]} \right)}}$$

Equation (12) is used for the excellent fits to the depolarization data in Fig. 5, and it predicts a plateau at a high [HY] of K_∞_ = *k*_1_*k*_d_/*k*_2_. Assuming *k*_1_/*k*_2_ is approximately constant for HCl, HBr, and DI, then *K*_∞_=1/τ_∞_ is proportional to *k*_d_, which is proportional to the hyperfine splitting of the complex and the quadrupole moment of the halogen nucleus. Indeed, it is observed that the high-pressure SPH lifetimes τ_∞_ = 40 ns for HCl, 16 ns for HBr, and 2.7 ns for DI scale approximately inversely proportional to the quadrupole moments of Cl, Br, and I, respectively^35^. These lifetimes of 3–40 ns are sufficient for the applications described in sections V-VII. In addition, it is predicted that much longer lifetimes are possible from the photodissociation of HF because the F nucleus (*I*_F_ = 1/2) lacks a quadrupole moment, and HF has hyperfine splittings approximately 2 orders of magnitude smaller than those of HCl.

### High-density SPH

When the 213 nm photodissociation laser pulse of ∼3 mJ is focused into the coil with a 50 mm lens, essentially all the HY molecules can be photodissociated, yielding SPH and Y atoms at the original HY density. In this case, the main SPH depolarization mechanism is from the collisions of SPH with Y atoms, as shown in Eq. (7). The depolarization cross section for SPH from halogen atoms is not in the literature. The SPH depolarization cross section from alkali atoms is approximately σ_alkali_ ∼2×10^−15^ cm^2^ ^37^. If we assume that the SPH depolarization cross section from halogens is also σ_halogen_ ∼2×10^−15^ cm^2^, then the rate constant *k*_Y_ = σ_halogen_ v, where v ∼ 2500 m/s is the relative velocity of the SPH and Y collisions, gives *k*_Y_ ∼ 5×10^−10^ cm^3^ s^**−**1^. Therefore, for a density [Y] = 10^19^ cm^−3^, the depolarization rate *k*_Y_[Y] = 5×10^9^ s^−1^, yielding an SPH lifetime of ∼0.2 ns. Under these conditions for DI, SPH lifetimes of approximately ∼10 ns longer were observed, and no evidence was seen for SPH depolarization from I(^2^P_3/2_). The authors concluded that the upper limit for the SPH depolarization cross section σ_halogen_ ≤ 10^−16^ cm^2^ (at least an order of magnitude smaller than σ_alkali_).

Another issue to consider is halogen atom recombination, which reduces SPH depolarization from halogen atoms, as well as the reaction of SPH with recombination product Y_2_, which removes SPH. The recombination and SPH reactions are

13 $$2{\mathrm{Y}} + {\mathrm{Y}}\, {\mathop \rightarrow \limits^{k_{r_1}}} \, {\mathrm{Y}}_2 + {\mathrm{Y}}$$

14 $$2{\mathrm{Y}} + {\mathrm{Y}}_2 \, {\mathop \rightarrow \limits^{k_{r_2}}} \, 2{\mathrm{Y}}_2$$

15 $${\mathrm{H}}^ \uparrow + {\mathrm{Y}}_2 \, {\mathop \rightarrow \limits^{k^{{\mathrm{Y}}_2}}} \, {\mathrm{HY}} + {\mathrm{Y}}$$

where $k_{r_2}$ = 3.1(3) × 10^−30^ cm^6^ molecule^−2^ s^−1^ for Y = I^38^, and $k_{r_1}$ = 3.5 × 10^−33^ e^810/T^ cm^6^ molecule^−2^ s^−1^ for Y = Cl^39^ (where T is the gas temperature); $k_{r_1}$ is generally smaller than $k_{r_2}$ (as Y is smaller and has fewer internal degrees of freedom than Y_2_), for example, $k_{r_1}$ = 8.75 × 10^−34^ e^810/T^ cm^6^ for Y = Cl^39^; for Y = Cl, $k^{{\mathrm{Y}}_2}$ = 1.83 ×10^−11^ cm^3^ molecule^−1^ s^−1^ at 298 K^40^, and $k^{{\mathrm{Y}}_2}$ = 6.3 × 10^−10^ cm^3^ molecule^−1^ s^−1^ at 730 K^41^.

We note that for Eq. (15), the reaction cross section of SPH with Cl_2_ ranges from ∼10^−16^ to ∼3×10^−15^ cm^2^ and is thus generally larger than the SPH-Cl depolarization cross section. This means that any reduction in SPH depolarization from reactions (13) and (14) is counterbalanced by reaction (15). Fortunately, there is no Y_2_ at t = 0, and recombination is slow enough to prevent significant Y_2_ formation at short times for all but the highest pressures. For example, for [Cl] = 10^20^ cm^−3^ at *T* = 298 K, the initial recombination rate $k_{r_1}$[Cl]^2^ ∼ 10^8^ s^−1^, showing that significant recombination occurs on timescales of approximately 10 ns and longer (similar times are found for [I] = 10^19^ cm^−3^).

Immediately after photodissociation, both the H and Y atoms are polarized, as shown by Eq. (1). However, in the Fourier transform trace of the polarization beating signal (e.g., in Fig. 4a), only hyperfine beatings from SPH are observable, and no signals are seen from the halogen atoms (for Cl, Br, or I). This indicates that the halogen atoms are depolarized very rapidly (for example, on the ∼1 ns scale or faster). The depolarization cross sections of Rb 5p(^2^P_3/2_) and Cs 6p(^2^P_3/2_) states from collisions with noble gas atoms have been shown to be on the order of 10^−14^ cm^2^ ^42,43^. If halogen atom (^2^P_3/2_)-state depolarization is also on the order of 10^−14^ cm^2^, then we expect rapid depolarization of the halogen atoms on the ∼40 ps timescale.

Therefore, the SPH lifetimes at “high density” of ∼10^19^ cm^−3^ and higher, in the presence of halogen atoms initially at similar density, are at least an order of magnitude longer than expected. The most plausible explanation is that the SPH halogen depolarization cross section is smaller than expected. Work is in progress to elucidate the SPH depolarization mechanism at “high density”, and recent work indicates an upper limit for the depolarization cross section σ_halogen_ ∼ 10^−16^ cm^2^ ^9,44^.

## Fast magnetometry

Conventional atomic magnetometers typically can operate down to ms timescales^45^, whereas magnetometers based on nitrogen-vacancy centres in diamond have recently been shown to operate down to μs timescales^46^. However, there are some applications that require ns or ps time resolution, such as the measurement of surface magnetism^47^, the generation of radical ion pairs in biochemical reactions such as photosynthesis^48^, and the ultrafast generation of magnetic fields with short-pulse lasers^49^, with applications in laser fusion, particle-beam generation, and laboratory astrophysics.

**Fig. 6:**
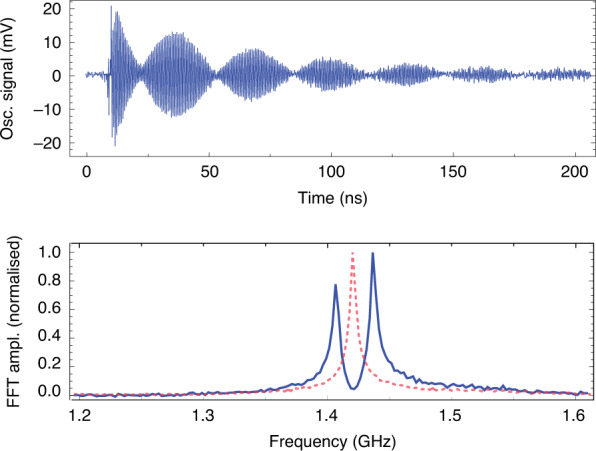
The Zeeman effect. (Top) Experimental SPH magnetization beating trace, with a transverse magnetic field of 10.76±0.01G. Notice the slow beating with a period of ∼30ns, caused by the beating of two frequencies. (Bottom) FFT of the top trace, showing that the trace (red dashed) without a magnetic field is split into two peaks (blue solid), due to the Zeeman effect. Clearly, this splitting, and the magnetic field strength, can be determined on the ns timescale

The SPH production and detection method can be used for fast magnetometry, as the magnetization beating frequency is sensitive to the magnetic field, and it can be measured on the ns timescale. The time-dependent magnetic field can be determined from analysis of the beating trace in the time domain. In addition, the average magnetic field over the signal decay time (from approximately 10–100 ns) can be determined by the Fourier transform of the signal. An example is shown in Fig. 6, where the hyperfine-beating peak at 1.42 GHz (for B = 0) is split into two peaks by a field of 10.76 G. Detailed demonstrations of such fast magnetometry measurements are given elsewhere^50^, including demonstrations of nanosecond-resolved measurements with sensitivities of ∼10 mG Hz^−1/2^ and proposals for achieving nanosecond-resolved measurements with sensitivities down to at least the μG scale.

## Laser-plasma acceleration

Polarized particle beams are used in scattering experiments in particle, nuclear, and solid-state physics. Conventional particle accelerators are typically very large and expensive facilities (often the size of ∼1 km, or larger, e.g., the LHC at CERN). In recent years, laser-plasma acceleration has allowed the production of MeV and GeV high-flux particle beams, accelerated over a distance of a few cm. However, these high-flux beams are produced from the laser acceleration of an unpolarized gas jet, yielding a non-spin-polarized beam.

**Fig. 7:**
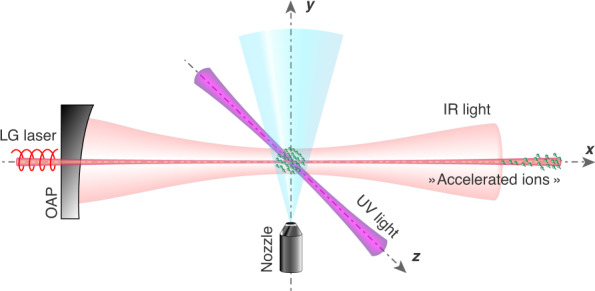
Experimental setup for laser acceleration of highly spin-polarized protons, deuterons ^52,53^, or electrons^10,51^. The nozzle produces an HY-molecule expansion in a vacuum chamber. An IR laser pulse aligns the HY bonds, and the circularly polarized UV laser photodissociates the HY molecules to produce SPH. The Y atoms can be removed by selective ionization with a UV laser. Finally, the spin-polarized protons, deuterons, or electrons from the SPH are ready for laser-driven wakefield acceleration by the high-power LG laser beam

Our group proposed that our high-density SPH source can be used for laser acceleration of polarized particles^9^, as it is the only source that provides spin-polarized electrons, protons, or deuterons at the densities needed for laser-plasma acceleration. Subsequently, Wen et al.^10^ and then Wu et al.^51^ proposed that, using SPH from HY photodissociation, spin-polarized electron beams can be produced from laser acceleration, with fluxes of order ∼1 kA, or approximately 4 orders of magnitude larger than those from conventional sources^10^. Furthermore, they presented calculations that showed that the polarization of the electrons would be reduced by less than 10% due to the laser acceleration process. A similar proposal was given by Hützen et al*.*^52^ for a laser-accelerated proton source, which also included experimental details needed to align the HY bonds with an IR laser to maximize the electron and nuclear polarization. Jin et al.^53^ theoretically showed that proton polarization will be largely maintained during laser acceleration. A schematic of the experimental setup is given in Fig. 7. Briefly, HY gas is expanded supersonically through a nozzle into a vacuum chamber, so that just below the nozzle, the desired HY density, in the 10^19^–10^20^ cm^−3^ range, is achieved. A focused infrared laser pulse aligns the HY bonds parallel to the axis of the linearly polarized IR light. At the peak of the IR pulse, when the HY molecules are fully aligned, a shorter, circularly polarized UV pulse photodissociates essentially all the HY molecules to produce SPH and halogen atoms. The halogen atoms can be removed by ionizing them with REMPI (for example, Cl(^2^P_3/2_) atoms ionize at 234.62 nm via 2+1 REMPI); the electrons leave first, and the Cl^+^ ions then leave the interaction region due to Coulomb explosion from the extremely high density of charge. The remaining high-density SPH atoms are used for laser-ion acceleration of spin-polarized electrons. In a similar setup, spin-polarized protons and deuterons can be accelerated; the removal of halogen atoms via ionization might not be necessary in this case because laser-accelerated protons or deuterons are separated from much heavier halogen nuclei. A review of the production of polarized beams from laser-plasma acceleration is given elsewhere^54^.

## Tests of polarized fusion

The development of nuclear fusion as an energy source has been pursued for many years to address the increasing demand for clean energy with a small environmental impact. The two main methods, magnetic confinement fusion (MCF) and inertial confinement fusion (ICF), are slowly approaching the break-even point, which needs to be exceeded for fusion to generate energy. Therefore, any contribution that can improve the efficiency of nuclear fusion can improve its viability as a commercial source of clean energy.

The three most important hydrogen isotope-based fusion reactions are deuterium–tritium (D–T), deuterium–helium-3 (D–^3^He), and D-D:

16 $${\mathrm{D}} + {\mathrm{T}} \to {^4\mathrm{He}} + {\mathrm{n}}$$

17 $${\mathrm{D}} + {^3\mathrm{He}} \to {^4\mathrm{He}} + {\mathrm{p}}$$

18A $${\mathrm{D}} + {\mathrm{D}} \to {^3\mathrm{He}} + {\mathrm{n}}$$

18B $${\mathrm{D}} + {\mathrm{D}} \to {\mathrm{T}} + {\mathrm{p}}$$

where D has a nuclear spin I_D_=1, and T and ^3^He have nuclear spins I_T_ = I_He-3_ = 1/2.

It has been known for several decades that polarizing the nuclear spins in the D–T and D–^3^He reactions, Eqs. (16) and (17), increases the fusion cross sections by 50% (verified from scattering experiments) and can also increase the reactor fusion yield by approximately 75%^55,56^. In contrast, it is not known what effect nuclear polarization has on the D-D reaction, as various difficult nuclear calculations are not in agreement, and no experiments have been performed^57^. In addition, it has not yet been demonstrated that nuclear polarization will survive long enough in the plasma to benefit nuclear fusion, although calculations indicate that it will for ICF^58^. In all these cases, the lack of tests of polarized fusion in a plasma stems from the inability of conventional methods to produce sufficient quantities of polarized D and T, as well as the difficulty in transporting and introducing the polarized fuel into a reactor.

An intriguing possibility is offered by the production of high-density SPD and SPT from hydrogen halide photodissociation, for a relatively straightforward way to perform a test of polarized fusion. SPD can be produced at high density in situ, along with spin-polarized ^3^He (or possibly SPT from TY photodissociation), and fusion events can be initiated with high-power lasers at laser-fusion facilities. The angular distribution D(θ,φ) of the neutron or proton products from the D–T or D–^3^He reactions is well-approximated by^9,59^

19 $$D\left( {\theta ,\varphi } \right) \approx \frac{{\sigma _0}}{{4\pi }}\left[ {\left( {2 + p} \right) - \left( {2p + p_{zz}} \right)P_2\left( {{\rm{cos}}\theta } \right)} \right]/3$$

where *p* = *p*_*z*_(D) *p*_*z*_ (W), W is T or ^3^He, *p*_*z*_ is the nuclear vector polarization, *p*_*zz*_ is the tensor polarization for D nuclei (here *p*_*zz*_ = 0), σ_0_ is the unpolarized fusion cross section, and P_2_(cos θ) is the second Legendre polynomial. Note that for *p* = 0, D(θ,φ) is isotropic (independent of θ and φ). For the D-^3^He reaction performed with *p*_*z*_(^3^He) = 0.8^60,61^ and *p*_*z*_(D) = ±0.12^9^ (the + sign is selected for σ^+^ circular photolysis light, and the − sign for σ^−^ light), giving p = ±0.1, and the angular distribution of the products is given by D(θ,φ) ∼1 ∓ (0.1)P_2_(cos θ); this has approximately a ±15% signal difference between product recoil directions of θ = 0° and 90°, and a ±5% difference in the integrated intensity. By employing DI bond alignment and increasing the D polarization by approximately a factor of 4 (giving *p*_*z*_(D) = ±0.5 and *p* = ±0.4), the signal effects are also increased by a similar factor. Similar experiments can likely be performed for D–T, replacing polarized ^3^He with polarized T from tritium halide photodissociation because the D–T fusion cross section is approximately 100 times larger than that for D-^3^He, yielding a much higher product yield. However, neither theory nor experiments have yet been performed on tritium halide photodissociation to verify that highly polarized T is produced.

The appeal of such tests of laser fusion is the simplicity of execution and that no further experimental development is needed. If it is shown that nuclear polarization survives ICF plasma and benefits fusion, then this may motivate the complicated development of nuclear spin-polarized fusion pellets, which are currently beyond the capabilities of conventional methods.

## Conclusions

SPH can be produced via the photodissociation of hydrogen halide molecules at extremely high densities of at least 10^19^ cm^−3^ and on very short timescales (fs-ns) using pulsed lasers (these densities and timescales are approximately 7 orders of magnitude higher and 10 orders of magnitude faster than conventional methods, respectively). The SPH depolarization times are surprisingly long, especially at high densities, as the depolarization cross section of SPH by halogen atoms seems to be smaller than expected. This new regime of high-density SPH opens up new applications for SPH, including laser-ion acceleration of polarized particles, ultrafast magnetometry, and tests of polarized nuclear fusion.

## Supplementary information

ACS publication permission for Fig. 5

IOP publication permission for Fig. 7

## References

[1] Steffens E, Haeberli W (2003). Polarized gas targets. Rep. Prog. Phys..

[2] Leader, E. *Spin in Particle Physics* (Cambridge University Press, 2001).

[3] Paetz gen. Schieck H (2010). The status of “polarized fusion”. Eur. Phys. J. A.

[4] Delgado F, Fernández-Rossier J (2017). Spin decoherence of magnetic atoms on surfaces. Prog. Surf. Sci..

[5] Leggett AJ (2001). Bose-Einstein condensation in the alkali gases: some fundamental concepts. Rev. Mod. Phys..

[6] Budker D, Romalis M (2007). Optical magnetometry. Nat. Phys..

[7] Ardenkjaer-Larsen JH (2015). Facing and overcoming sensitivity challenges in biomolecular NMR spectroscopy. Angew. Chem. Int. Ed..

[8] Oros AM, Shah NJ (2004). Hyperpolarized xenon in NMR and MRI. Phys. Med. Biol..

[9] Sofikitis D (2018). Ultrahigh-density spin-polarized H and D observed via magnetization quantum beats. Phys. Rev. Lett..

[10] Wen M, Tamburini M, Keitel CH (2019). Polarized laser-WakeField-accelerated Kiloampere electron beams. Phys. Rev. Lett..

[11] Szczerba D (2000). A polarized hydrogen/deuterium atomic beam source for internal target experiments. Nucl. Instrum. Methods Phys. Res. A: Accelerators, Spectrometers, Detect. Associated Equip..

[12] Clasie B (2006). Laser-driven target of high-density nuclear-polarized hydrogen gas. Phys. Rev. A.

[13] Redsun SG (1990). Production of highly spin-polarized atomic hydrogen and deuterium by spin-exchange optical pumping. Phys. Rev. A.

[14] Eppink ATJB (1998). Production of maximally aligned O(^1^D) atoms from two-step photodissociation of molecular oxygen. J. Chem. Phys..

[15] Rakitzis TP, Kitsopoulos TN (2002). Measurement of Cl and Br photofragment alignment using slice imaging. J. Chem. Phys..

[16] Chestakov DA (2006). Photofragment alignment in the photodissociation of I_2_ from 450 to 510 nm. J. Chem. Phys..

[17] Van Brunt RJ, Zare RN (1968). Polarization of atomic fluorescence excited by molecular dissociation. J. Chem. Phys..

[18] Vasyutinskiǐ OS (1980). Orientation of atoms during photodissociation of molecules. JETP Lett..

[19] Rakitzis TP (2003). Spin-polarized hydrogen atoms from molecular Photodissociation. Science.

[20] Rakitzis TP (2004). Pulsed-laser production and detection of spin-polarized hydrogen atoms. ChemPhysChem.

[21] Rakitzis TP (2004). Measurement of Br photofragment orientation and alignment from HBr photodissociation: production of highly spin-polarized hydrogen atoms. J. Chem. Phys..

[22] Rubio-Lago L (2006). Laser preparation of spin-polarized atoms from molecular photodissociation. Phys. Scr..

[23] Sofikitis D (2008). Nanosecond control and high-density production of spin-polarized hydrogen atoms. EPL (Europhys. Lett.).

[24] Sofikitis D (2008). Laser detection of spin-polarized hydrogen from HCl and HBr photodissociation: Comparison of H- and halogen-atom polarizations. J. Chem. Phys..

[25] Bougas L (2010). (2 + 1) laser-induced fluorescence of spin-polarized hydrogen atoms. J. Chem. Phys..

[26] Broderick BM (2013). Velocity distribution of hydrogen atom spin polarization. J. Phys. Chem. Lett..

[27] Broderick BM (2014). Spin-polarized hydrogen Rydberg time-of-flight: experimental measurement of the velocity-dependent H atom spin-polarization. Rev. Sci. Instrum..

[28] Broderick BM (2015). Imaging detection of spin-polarized hydrogen atoms. Chem. Phys. Lett..

[29] Sofikitis D (2017). Highly nuclear-spin-polarized deuterium atoms from the UV photodissociation of deuterium Iodide. Phys. Rev. Lett..

[30] Balint-Kurti GG (2002). Vector correlations and alignment parameters in the photodissociation of HF and DF. J. Chem. Phys..

[31] Brown A, Balint-Kurti GG, Vasyutinskii OS (2004). Photodissociation of HCL and DCL: polarization of atomic photofragments. J. Phys. Chem. A.

[32] Brown A (2005). Photodissociation of HI and DI: polarization of atomic photofragments. J. Chem. Phys..

[33] Jodoin DN, Brown A (2005). Photodissociation of HI and DI: testing models for electronic structure via polarization of atomic photofragments. J. Chem. Phys..

[34] Sofikitis D (2019). Photofragment spin-polarization measurements *via* magnetization quantum beats: dynamics of DI photodissociation. Phys. Chem. Chem. Phys..

[35] Boulogiannis GK (2019). Spin-polarized hydrogen depolarization rates at high hydrogen halide pressures: hyperfine depolarization via the HY-H complex. J. Phys. Chem. A.

[36] Milner AA, Korobenko A, Milner V (2017). Ultrafast magnetization of a dense molecular gas with an optical centrifuge. Phys. Rev. Lett..

[37] Zelenski AN, Kokhanovski SA (1993). A study of spin‐exchange polarization transfer in hydrogen‐rubidium collisions. AIP Conf. Proc..

[38] Ip JKK, Burns G (1972). Recombination of iodine atoms by flash photolysis over a wide temperature range. II I_2_ in He, Ar, Xe, N_2_, CO. J. Chem. Phys..

[39] Kemaneci E (2014). Global (volume-averaged) model of inductively coupled chlorine plasma: influence of Cl wall recombination and external heating on continuous and pulse-modulated plasmas. Plasma Sources Sci. Technol..

[40] Bykhalo IB (1994). Kinetics study of two-channel hydrogen and deuterium atom reactions with interhalogen molecules. Russian Chem. Bull..

[41] Baulch DL (1981). Evaluated kinetic data for high temperature reactions. Volume 4: homogeneous gas phase reactions of halogen- and cyanide-containing species. J. Phys. Chem. Ref. Data.

[42] Gallagher A (1967). Collisional depolarization of the Rb 5*p* and Cs 6*p* doublets. Phys. Rev..

[43] Bayram SB (2006). Collisional depolarization of Zeeman coherences in the ^133^Cs 6*p*^2^*P*_3/2_ level: double-resonance two-photon polarization spectroscopy. Phys. Rev. A.

[44] Spiliotis, A. K. et al. Depolarization of spin-polarized hydrogen via spin-exchange collisions with chlorine atoms at ultrahigh density. Preprint at https://arxiv.org/abs/2101.02675 (2021).

[45] Jiménez-Martínez R (2012). High-bandwidth optical magnetometer. J. Optical Soc. Am. B.

[46] Zheng HJ (2020). Microwave-free vector magnetometry with nitrogen-vacancy centers along a single axis in diamond. Phys. Rev. Appl..

[47] Sirotti F (1998). Time-resolved surface magnetometry in the nanosecond scale using synchrotron radiation. J. Appl. Phys..

[48] Link G (2004). High time resolution multifrequency EPR of radical pair intermediates in photosynthetic reaction centers: Structure determination on a nanosecond time scale. Z. Physik. Chem..

[49] Zhu BJ (2018). Ultrafast pulsed magnetic fields generated by a femtosecond laser. Appl. Phys. Lett..

[50] Spiliotis, A. K. et al. A nanosecond-resolved atomic hydrogen magnetometer. Preprint at https://arxiv.org/abs/2010.14579 (2020). 10.1039/d1cp03171f 34549209

[51] Wu YT (2019). Polarized electron-beam acceleration driven by vortex laser pulses. N. J. Phys..

[52] Hützen A (2019). Polarized proton beams from laser-induced plasmas. High Power Laser Sci. Eng..

[53] Jin LL (2020). Spin-polarized proton beam generation from gas-jet targets by intense laser pulses. Phys. Rev. E.

[54] Büscher M (2020). Polarized beams from laser-plasma accelerators. High. Power Laser Sci. Eng..

[55] Temporal M (2012). Ramis. Ignition conditions for inertial confinement fusion targets with a nuclear spin-polarized DT fuel. Nucl. Fusion.

[56] Hupin G, Quaglioni S, Navrátil P (2019). Ab initio predictions for polarized deuterium-tritium thermonuclear fusion. Nat. Commun..

[57] Engels R (2014). Polarized fusion. Phys. Part. Nucl..

[58] More RM (1983). Nuclear spin-polarized fuel in inertial fusion. Phys. Rev. Lett..

[59] Kulsrud RM (1982). Fusion reactor plasmas with polarized nuclei. Phys. Rev. Lett..

[60] Batz M (2005). ^3^He spin filter for neutrons. J. Res. Natl Inst. Stand. Technol..

[61] Walker TG, Happer W (1997). Spin-exchange optical pumping of noble-gas nuclei. Rev. Mod. Phys..

