# Supplementary material for: Ultrahigh-density spin-polarized hydrogen isotopes from the photodissociation of hydrogen halides: new applications for laser-ion acceleration, magnetometry, and polarized nuclear fusion
Source: Light Sci Appl. 2021 Feb 12;10:35. doi: 10.1038/s41377-021-00476-y (PMC7881141; doi:10.1038/s41377-021-00476-y)
Supplement: Supplementary file 1 — ACS publication permission for Fig. 5 [file 41377_2021_476_MOESM1_ESM.pdf]

Spin-Polarized Hydrogen Depolarization Rates at High Hydrogen Halide Pressures: Hyperfine Depolarization via the HY–H Complex

Author: Gregoris K. Boulogiannis, Chrysovalantis S. Kannis, Georgios E. Katsoprinakis, et al

Publication: The Journal of Physical Chemistry A

Publisher: American Chemical Society

Date: Sep 1, 2019

Copyright © 2019, American Chemical Society

PERMISSION/LICENSE IS GRANTED FOR YOUR ORDER AT NO CHARGE

This type of permission/license, instead of the standard Terms & Conditions, is sent to you because no fee is being charged for your order. Please note the following:

- Permission is granted for your request in both print and electronic formats, and translations.
- If figures and/or tables were requested, they may be adapted or used in part.
- Please print this page for your records and send a copy of it to your publisher/graduate school.
- Appropriate credit for the requested material should be given as follows: "Reprinted (adapted) with permission from (COMPLETE REFERENCE CITATION). Copyright (YEAR) American Chemical Society." Insert appropriate information in place of the capitalized words.
- One-time permission is granted only for the use specified in your request. No additional uses are granted (such as derivative works or other editions). For any other uses, please submit a new request.

If credit is given to another source for the material you requested, permission must be obtained from that source.

BACK

CLOSE WINDOW
