# Supplementary material for: Ultrahigh-density spin-polarized hydrogen isotopes from the photodissociation of hydrogen halides: new applications for laser-ion acceleration, magnetometry, and polarized nuclear fusion
Source: Light Sci Appl. 2021 Feb 12;10:35. doi: 10.1038/s41377-021-00476-y (PMC7881141; doi:10.1038/s41377-021-00476-y)
Supplement: Supplementary file 2 — IOP publication permission for Fig. 7 [file 41377_2021_476_MOESM2_ESM.pdf]

Attribution 3.0 Unported (CC BY 3.0)

This is a human-readable summary of (and not a substitute for) the license. Disclaimer.

You are free to:

Share — copy and redistribute the material in any medium or format

Adapt — remix, transform, and build upon the material for any purpose, even commercially.

The licensor cannot revoke these freedoms as long as you follow the license terms.

Under the following terms:

Attribution — You must give appropriate credit, provide a link to the license, and indicate if changes were made. You may do so in any reasonable manner, but not in any way that suggests the licensor endorses you or your use.

No additional restrictions — You may not apply legal terms or technological measures that legally restrict others from doing anything the license permits.
